# Special status of iron transfer proteins in the serum of breast cancer patients

**DOI:** 10.1007/s12672-025-02799-3

**Published:** 2025-05-31

**Authors:** Lama Alhaddad, Maher Saifo, Ranwa Alsayed

**Affiliations:** 1 Department of Biochemistry and Microbiology, Faculty of Pharmacy, Damascus University, Damascus, Syrian Arab Republic; 2 Department of Oncology, Damascus University, Damascus, Syrian Arab Republic

**Keywords:** Breast cancer, Transferrin, Lipocalin 2, Estrogen receptor, Progesterone receptor, Syria

## Abstract

Cancer cells have a greater requirement for iron because of their continuous and rapid proliferation. To do that, iron homeostasis is altered in cancer cells to meet this excessive demand. Transferrin (TF) and lipocalin 2 (LCN2) are proteins that play essential roles in transporting and delivering iron into cells. The aim of this study was to investigate the serum levels of transferrin and lipocalin 2 in Syrian breast cancer patients, and compare them with those in a control group and their correlation with the tumor state and characteristics. This case‒control study included 80 Syrian women divided into two groups: a group of patients with breast cancer who were newly diagnosed via biopsy (n = 40), and a control group of healthy women (n = 40). Serum transferrin levels were measured via an immunoturbidimetric assay and serum levels of lipocalin 2 were measured via an enzyme-linked immunosorbent assay (ELISA). Compared with the control group, the case group presented lower levels of Lipocalin 2, but serum LCN2 levels were correlated with estrogen and progesterone receptor (ER,PR) negative status (r = -0.330, *p*-value = 0.038) and (r = -0.441, *p*-value = 0.004) respectively. However, transferrin levels were correlated only with progesterone receptor status. Interestingly, there was a negative correlation between serum lipocalin 2 and transferrin serum levels (r = -0.416, *p*-value = 0.008). This study revealed that serum lipocalin 2 levels decrease in breast cancer patients with increased tumor expression of the ER and PR receptors, it also indicates the collaboration and the cross-talk of iron transport proteins to deliver it to cancer cells. This suggests the importance of iron transport proteins as novel biomarkers and perhaps as therapeutic targets for aggressive subtypes of breast cancer (ER-negative and PR-negative subtypes).

## Introduction

Breast cancer is the most common cancer diagnosed in women, accounting for more than 1 in 10 new cancer diagnoses each year. It is also the second most common cause of cancer death among women around the world [1]. In 2022, there were 2.3 million women diagnosed with breast cancer and 670 000 deaths globally [2].

In Syria, breast cancer is the most common cancer, accounting for nearly 35% of all new cancer cases, and is the leading cause of cancer death as of 2022 [3].

Breast cancer is divided into multiple subtypes. One of the accepted classifications of breast cancer is based on the expression of the hormone receptors: estrogen receptor positive (ER +), progesterone receptor positive (PR +), and human epidermal growth factor receptor positive (HER2 +). These receptors are considered diagnostic and prognostic biomarkers of breast cancer. HER2 overexpression is often an early sign of breast cancer [4], and may be associated with histological grade II and ductal carcinomas [5].

Many biochemical markers have been examined in breast tumors, most of them relate to reactive oxygen species (ROS) production which can stimulate modifications to DNA, proteins and lipids. ROS leads to cancer cell metabolism being directed toward “Warburg effect", which induces migration and invasion and promotes a more aggressive tumor phenotype [6].

Accordingly, recent studies have moved towards antioxidants such as Lactate dehydrogenase (LDH), superoxide dismutase (SOD) and catalase (CAT) [7], and towards stimulants of ROS production in cancer cells, such as iron, as its excess leads to the production of ROS via the Fenton reaction [8].

Since iron may be one of the causes of oxidative stress in the cell, in addition to being an essential nutrient, and a cofactor for enzymes involved in DNA replication and energy production, the need for iron increases in the case of rapidly dividing cells, and it is important to study the variability of its metabolism in cancer cases, especially the variations of proteins related to its transfer to the cells, transferrin as the major transporter and lipocalin 2 [6]. A previous review [9] indicated the important role of iron in carcinogenesis and the link between the dysregulations of proteins related to its metabolism and cancer patient prognosis.

Transferrin is a circulating glycoprotein that acts as an iron transporter; it is an important biomarker that reflects the iron status in the body. In addition, malignancy has been identified as one of the causes of low transferrin levels [10].

Another important biomarker is lipocalin-2 (LCN2), also called neutrophil gelatinase-associated lipocalin (NGAL), which is a secreted glycoprotein belongs to the adipokine superfamily. This protein involves in intracellular iron homeostasis using siderophore-like molecules, such as catechol [11]. According to a previous review [12], recently, attention has turned to LCN2 as a new biomarker and modulator of breast cancer.

Several studies have also reviewed in the two previous reviews [11, 12] and have indicated that the tissue and serum levels of LCN2 are associated with the severity of cancer, recurrence, and poor prognosis in patients, and this may be due to the role of this protein in trafficking iron into the cells.

In this study we evaluated the serum levels of transferrin and lipocalin 2 in Syrian breast cancer patients and compared them with those in a control group, and we also studied the correlation of their levels with the tumor status in patients.

## Subjects and methods

### Subjects

Patients with breast cancer were recruited from the Albayrouni University Hospital, Damascus, Syria.

Controls were recruited from the National University Hospital, Damascus, Syria. The recruitment phase was between December 2023 and August 2024. We enrolled 80 Syrian females in this study, including 40 with breast cancer and 40 healthy controls.

The breast cancer patients (n = 40) were newly diagnosed and had histologically confirmed breast cancer with no prior surgical, chemotherapy, or radiotherapy treatment for breast cancer, and who attended the Albayrouni University Hospital between December 2023 and April 2024.

The healthy control subjects (n = 40) were matched for age and body mass index (BMI) with the breast cancer group, and they were confirmed to be free from benign or malignant breast diseases through mammography.

The exclusion criteria for all participants were history of other tumors, consumption of iron supplements and oral contraceptives, iron deficiency anemia, kidney or liver disease or other chronic diseases and history of recent blood transfusion.

For all subjects, comprehensive questionnaires were used. A complete history was obtained, including lifestyle behaviors, medical history, menstrual and reproductive history, and menopausal status, as well as a family history of breast cancer and other cancers.

For the patient group, information about the patient’s cancer status including the data for hormonal receptor status was obtained by referring to each patient’s hospital file; The assessment of hormonal receptors status is routinely performed by a pathologist in the pathological anatomy laboratory in the hospital using an immunohistochemical based technique for each patient as previously shown in other works [13].

### Ethics statement

The study protocol was approved by the Biomedical Research Ethics Committee (BMREC) at Damascus University (12/2/2024) (ID number: PH-120224-194) in accordance with the Declaration of Helsinki (1964). Participants were fully informed of the aims of the study and provided written informed consent for their participation.

### Blood sampling and biochemical measurements

Venous blood (5 ml) was collected into dry tubes, and the tubes were chilled on ice and centrifuged within 1 h. Several aliquots were prepared and stored at − 20°C for subsequent assays. Serum transferrin levels were measured via immunoturbidimetric assay via a kit from the Swiss company Roche Cobas according to the manufacturer’s instructions.

Serum levels of lipocalin 2 were measured via sandwich enzyme-linked immunosorbent assay (ELISA) via kit from the Chinese company Sunlong Biotech according to the manufacturer’s instructions as suggested in other works [14]. The resulting color was read at 450 nm using the wavelength 600 nm as reference wavelength. Then, the value of lipocalin 2 was calculated, estimated in nanograms\Milliliter (ng\ml).

### Statistical analysis

The statistical analyses were performed with SPSS (version 26.0). The results are shown as the means (SD, standard deviation). Continuous variables were compared between two or three independent groups via ANOVA or the Mann–Whitney test, respectively. The chi-square test was applied to compare categorical variables. Correlation between variables was studied via the Spearman test. P values less than 0.05 were considered statistically significant.

## Results

### Study population characterization

Table 1 shows the main characteristics of the study population. Patients and controls were similar in terms of age and BMI distribution. There was no statistically significant difference between the two groups in terms of smoking status or marital status, but the percentage of Viviparous women was greater in the control group (*p*-value = 0.037). (Table 1).

**Table 1:** Main characteristics of the study population

	Case N = 40	Control N = 40	*p*-value
Age (years)	51.7 (8.3)	51.6 (8.4)	0.668
BMI (kg/m^2^)	28 (6.5)	29 (5)	0.247
Smoker (%)			
Yes	22.5	30	0.612
No	77.5	70	
Marital status			
Married	77.5	87.5	0.593
Single	15	7.5	
Divorced or widowed	7.5	5	
Viviparous			
Yes	72.5	92.5	0.037
No	27.5	7.5	

Data are means (SD) unless otherwise specified. Differences between medians of the continuous variables were tested using Mann Whitney U test*.* Chi-square test was used to compare the categorical variables. *p* < 0.05 was considered statistically significant

### Differences between the two study groups in terms of the serum levels of transferrin and lipocalin 2

The serum levels of transferrin and lipocalin 2 of breast cancer patients and controls are depicted in Table 2. Compared with the control women, women with breast cancer presented lower serum levels of lipocalin 2 (mean 60.7 vs. 74.8 ng/ml; *p* = 0.004). The transferrin serum levels did not differ between the two groups. (Table 2).

**Table 2:** Serum levels of iron, Transferrin and Lipocalin 2 of breast cancer patients and controls

	Case N = 40	Control N = 40	*p*-value
Transferrin TF (mg\dl)	271 (46.5)	284.65 (40.7)	0.285
Lipocalin 2 LCN2 (ng\ml)	60.7 (32.4)	74.8 (22.9)	0.004

Data are means (SD)

^*^Mean Ranks were compared using Mann Whitney U test. *p* < 0.05 was considered statistically significant

### Correlations between LCN2, breast cancer receptor status and transferrin serum levels

Spearman rank correlation analysis, as shown in Table 3, revealed that serum LCN2 levels were correlated with estrogen and progesterone receptor negative status (r =—0.330, *p*-value = 0.038) and (r =—0.441, *p*-value = 0.004) respectively. However, there was no correlation between the serum levels of lipocalin 2 or transferrin and grade, lymph node status, tumor size, or metastasis. Notably, transferrin levels were significantly higher in patients with positive progesterone receptor expression.

**Table 3:** Spearman rank correlation coefficients of the study variables in the patients

Grade		Tumor size T	Lymph node state N	Distant metastasis M	TNM staging	ER	PR	HER 2
LCN2 (ng\ml)								
R	0.180	0.221	0.193	-0.038	0.036	-0.330	-0.441	0.202
* p*-value	0.266	0.171	0.233	0.817	0.827	0.038*	0.004**	0.211
N	40	40	40	40	40	40	40	40
TF (mg\dl)								
R	−0.089	−0.070	-0.107	0.241	0.138	0.046	0.342	-0.066
* p*-value	0.585	0.668	0.510	0.134	0.397	0.780	0.031*	0.686
N	40	40	40	40	40	40	40	40

*LCN2* Lipocalin 2, *TF* transferrin, *r* correlation coefficient, *ER* estrogen receptor,* PR* progesterone receptor, *HER2* human epidermal growth factor receptor 2

^*^ Correlation is significant at the 0.05 level (2-tailed)

^**^Correlation is significant at the 0.01 level (2-tailed). *p* < 0.05 was considered statistically significant

**Fig. 1:**
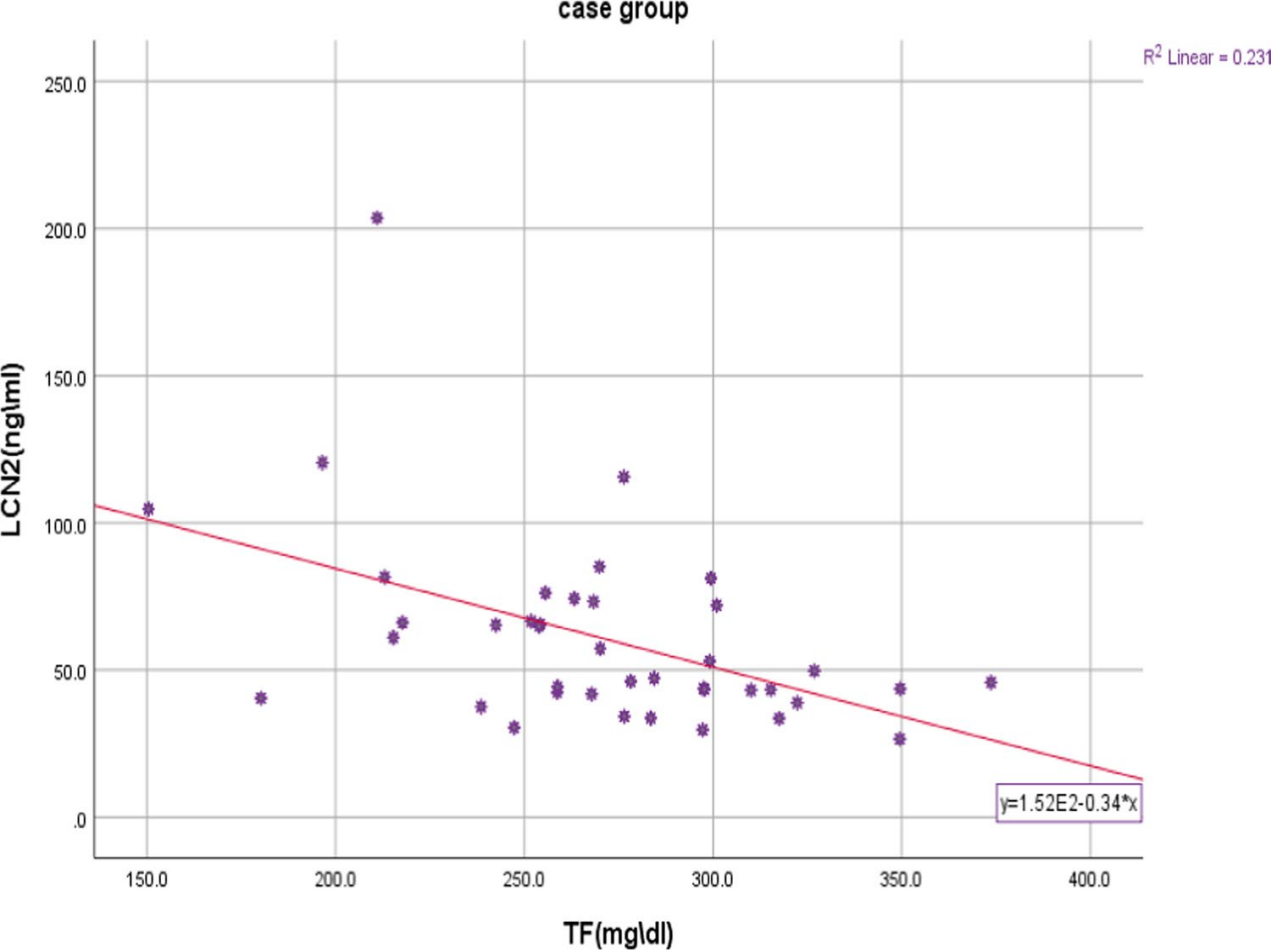
The correlation between serum Lipocalin 2 and Transferrin levels in the patient group. The correlation coefficients were determined via the Spearman test. *p*-value = 0.008, r = − 0.416. *LCN2* Lipocalin 2, *TF* Transferrin

Another significant negative correlation was found between LCN2 and transferrin (TF) serum levels (r =—0.416, *p*-value = 0.008). (Fig. 1).

**Fig. 2:**
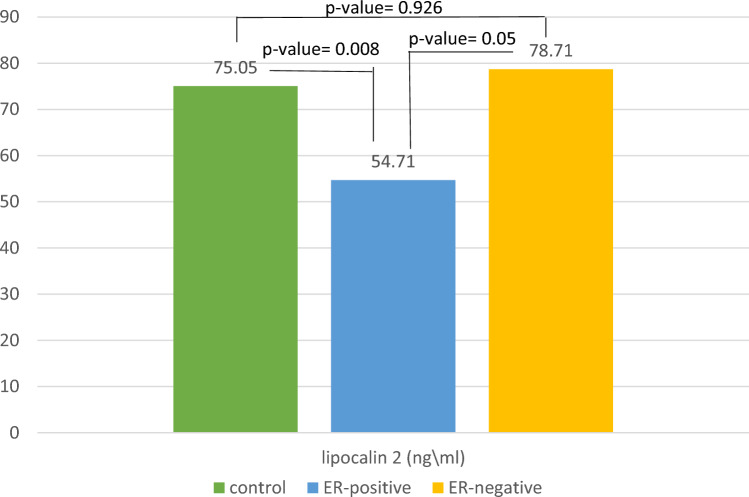
The difference between three subgroups (control, patient with ER- tumor, and patients with ER + tumor) in terms of Serum levels of Lipocalin 2. *p*-value between the three subgroups was 0.006. Means were compared using ANOVA test followed by Tukey HSD test.* p* < 0.05 was considered statistically significant

However, there was no correlation between LCN2 and transferrin (TF) serum levels in the control group (r = 0, *p*-value = 1). (Fig. 2).

### Differences among the three subgroups (controls, patients with ER-negative tumors, and patients with ER-positive tumors) in terms of serum levels of Lipocalin 2 were determined via ANOVA test followed by Tukey’s HSD test

The means concentrations of Lipocalin 2 in the three subgroups are depicted and compared in Fig. 2. The group of patients with ER-negative tumors had a greater mean concentration of lipocalin 2 than did the other two groups did. Compared with controls, patients with ER-positive tumors presented lower concentrations of LCN2 (54.7 vs. 75.07; *p* = 0.008), but the concentrations of LCN2 in patients with ER-negative tumors tended to be greater than those in the ER-positive subgroup (78.7 vs. 54.7; *p* = 0.05). The serum levels of LCN2 did not differ between the ER-negative subgroup and the controls.

### Sensitivity and specificity of serum lipocalin 2 levels

**Fig. 3:**
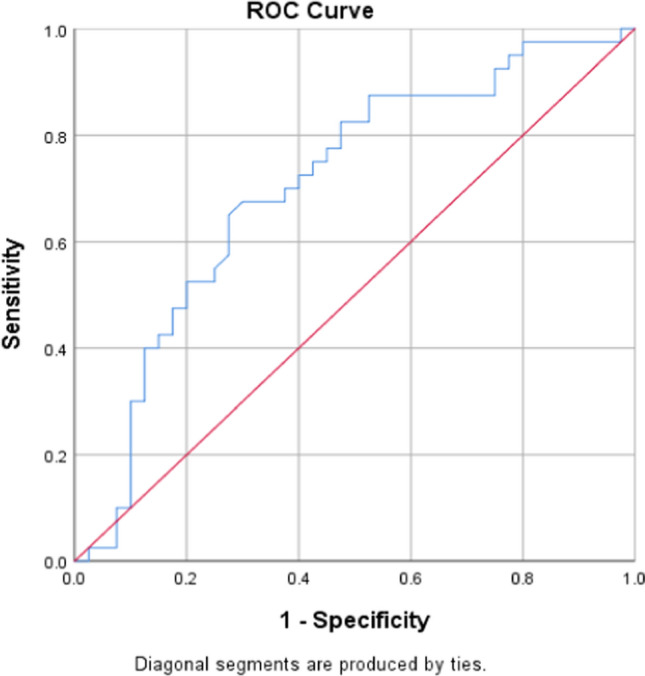
ROC curve for serum lipocalin 2 levels

The sensitivity and specificity of serum lipocalin 2 levels were studied using the receiver operating characteristic curve (ROC Curve) test, and the value of 81.5 ng/ml was determined as a threshold value at which the sensitivity and specificity were the highest possible (40% and 73%, respectively) (Fig. 3), and the area under the curve was also calculated (Table 4).

**Table 4:** Area under the curve description

Area under the curve	Standard error	*p*-value
0.706	0.059	0.002

## Discussion

Many studies have addressed the metabolic disorders in breast tumors, such as glycolytic metabolism of carbohydrates even in the presence of oxygen (anerobic glycolysis) and related enzymes which are important and in clinical use [15], and altered iron metabolism and the dysregulation of the proteins related to it as prognostic biomarkers for breast cancer [9].

In this study, the serum levels of transferrin and lipocalin 2 were evaluated and compared between patients with breast cancer and healthy women. In addition, we studied the associations of these biomarkers with the tumor status of patients. Transferrin serum levels did not differ between the patient and control groups. However, higher serum concentrations of LCN2 are observed in heathy women than in women with breast cancer. Interestingly, the serum LCN2 concentration decreases as tumor cells express estrogen and progesterone receptors, and higher levels of LCN2 correlate with an ER-negative status.

Our results support the findings of a previous study conducted in mice, as Drew et al. [16] reported that the estrogen receptor ERα binds to the LCN2 promoter and competes with the transcription factor CCAAT/enhancer-binding protein (C/EBP) to regulate the expression of LCN2, leading to repression of its expression.

Because Cytokines are secreted by the tumor microinveronment (TME), stimulating the transcription of LCN2 by inducing the activation and binding of transcription factors such as CCAAT/enhancer-binding protein (C/EBP) to the LCN2 promoter region [11], high levels of lipocalin 2 associated with the ER-negative, PR-negative subtype of breast cancer may also be explained by the fact that several studies have demonstrated an increase in inflammatory infiltrates in the subtype of hormone receptor-negative breast cancer [17]. In the same context, ER + breast cancer cells express and/or secrete lower cytokine levels than ER- cells do [18]. Similarly, several reviews have revealed the associations of high levels of lipocalin 2 with poor prognosis in ER − /PR − /HER2 + tumors, and ER- and PR-negative status in tumor samples from patients with breast cancer [11, 12], which is consistent with our study. ROC curve for lipocalin 2 indicates low diagnostic value in the general breast cancer group; as discussed previously, the serum levels of lipocalin 2 differed between patients of different subgroups, which may indicate important prognostic rather than diagnostic value.

Finally, in the patient group, a negative correlation was found between the concentrations of lipocalin 2 and transferrin in the serum, wherease there was no correlation between them in healthy women. That is, when the concentration of transferrin as the major iron transporter protein [6] in the blood of breast cancer patients decreases, the concentration of lipocaline 2 in their blood increases as an alternative mechanism to meet the demand of cancer cells for iron, which cannot be mediated solely via the Tf-TfR-mediated pathway. Therefore, Lcn-2, a transferrin-independent iron carrier, which tumor-associated macrophages (M2-phenotype) increase its expression in the more aggressive tumor environment, allows cancer cells to acquire the additional necessary iron [19].

The current study has few limitations. First, small sample size. Second, evaluating transferrin as a protein without calculating its saturation. Third, the inability to follow up on patients. Finally, lack of more data regarding breast tumor markers such as CA 15-13 and CA 27.29.

## Conclusions and recommendations

Taken together, the results of the present study revealed differences in the serum levels of several proteins related to iron metabolism, such as lipocalin 2, between breast cancer patients and healthy controls. This finding also indicates that higher serum levels of lipocalin 2 in breast cancer patients are associated with an aggressive subtype of breast cancer (ER-and PR-negative subtypes).

Because current treatment options for aggressive cancers are limited, These proteins may be promising therapeutic targets against these cancer subtypes.

This research area is still under development, and further clinical studies with a larger number of samples are necessary to study the heterogeneity of iron metabolism between breast cancer subtypes.

We also recommend that patients be followed up to investigate the prognostic value of lipocalin 2 in breast cancer patients, which is essential to determine its potential as a future therapeutic target for some aggressive subtypes of breast cancer.

## Data Availability

All data generated or analysed during this study are included in this published article.
